# Genetic Analysis of the Roles of BMP2, BMP4, and BMP7 in Limb Patterning and Skeletogenesis

**DOI:** 10.1371/journal.pgen.0020216

**Published:** 2006-12-22

**Authors:** Amitabha Bandyopadhyay, Kunikazu Tsuji, Karen Cox, Brian D Harfe, Vicki Rosen, Clifford J Tabin

**Affiliations:** 1 Department of Genetics, Harvard Medical School, Boston, Massachusetts, United States of America; 2 Department of Developmental Biology, Harvard School of Dental Medicine, Boston, Massachusetts, United States of America; 3 Department of Molecular Genetics and Microbiology, University of Florida, Gainesville, Florida, United States of America; University of Pennsylvania School of Medicine, United States of America

## Abstract

Bone morphogenetic protein (BMP) family members, including BMP2, BMP4, and BMP7, are expressed throughout limb development. BMPs have been implicated in early limb patterning as well as in the process of skeletogenesis. However, due to complications associated with early embryonic lethality, particularly for *Bmp2* and *Bmp4*, and with functional redundancy among BMP molecules, it has been difficult to decipher the specific roles of these BMP molecules during different stages of limb development. To circumvent these issues, we have constructed a series of mouse strains lacking one or more of these BMPs, using conditional alleles in the case of *Bmp2* and *Bmp4* to remove them specifically from the limb bud mesenchyme. Contrary to earlier suggestions, our results indicate that BMPs neither act as secondary signals downstream of Sonic Hedghog (SHH) in patterning the anteroposterior axis nor as signals from the interdigital mesenchyme in specifying digit identity. We do find that a threshold level of BMP signaling is required for the onset of chondrogenesis, and hence some chondrogenic condensations fail to form in limbs deficient in both BMP2 and BMP4. However, in the condensations that do form, subsequent chondrogenic differentiation proceeds normally even in the absence of BMP2 and BMP7 or BMP2 and BMP4. In contrast, we find that the loss of both BMP2 and BMP4 results in a severe impairment of osteogenesis.

## Synopsis

A group of related signaling molecules called bone morphogenetic proteins (BMPs) are known to play important roles in the formation of the structures such as the limbs. However, because different members of this group often have similar effects on target cells and are produced in overlapping regions of the embryo and hence can be redundant with one another, removal of any single member of the BMP family may not reveal the full extent of the roles they play during development. We have therefore improved on this type of analysis by removing pairs of these factors (BMP2 and BMP4 or BMP2 and BMP7) specifically from the developing limb. Although some have speculated that these signals play an early role in organizing or “patterning” the different tissues of the limb, we find no evidence for such a role. We do find, however, that a minimal amount of BMP signal is required to form cartilage, and hence some cartilaginous elements fail to form in limbs deficient in both BMP2 and BMP4. Moreover, in the absence of these two BMP family members, there is a severe impairment in the development of bone tissue, resulting in severely deformed limbs. This study gives important new insight into the roles of these BMP signals in making skeletal tissues in the embryo.

## Introduction

Bone morphogenetic proteins (BMPs) are secreted signaling molecules belonging to the transforming growth factor β superfamily, originally identified on the basis of their ability to induce ectopic bone formation when implanted within soft tissue in vivo [1–3]. BMP family members are now known to play an extremely diverse set of roles in a wide variety of developmental processes [4]. Even in the context of the morphogenesis of a single structure, these molecules can play a series of quite divergent roles. For example, during limb development, BMPs have been postulated to act sequentially in multiple distinct aspects of patterning, cell type specification, and differentiation of various tissues, particularly of the skeleton.

The earliest step of limb development in which BMP signaling has been implicated is the establishment of the anterior-posterior limb axis. Differences in anterior-posterior pattern are instructed as a graded response to Sonic Hedghog (SHH) signaling emanating from the posterior margin of the limb bud [5]. It has remained controversial, however, whether this response is direct or indirect. If indeed the long-range effects of SHH are indirectly mediated by local production of secondary signals, the leading candidates have been two members of the BMP family, BMP2 and BMP7. Both are expressed in a broader domain than SHH in the early posterior limb bud mesenchyme [6,7], although BMP7 also has a second, weaker domain of expression in the anterior limb bud mesenchyme. BMP2 [8] and BMP7 [7] can be induced by ectopic SHH and their expression is greatly diminished in the absence of SHH activity [9]. BMP2 and BMP7 are thus secondary signals produced in response to SHH activity. Moreover, BMP signaling has a weak ability to posteriorly polarize the limb in ectopic grafting experiments [10], an activity enhanced by prior low-level exposure to SHH [11]. It remains unclear, however, whether BMP2 and BMP7 activity is required endogenously for anterior-posterior limb patterning by SHH. *Bmp2* mutant embryos die too early to assess their limb phenotypes. A targeted deletion of *Bmp7* has been made, and *Bmp7*-deficient embryos display hindlimb polydactyly with incomplete penetrance but otherwise phenotypically normal limbs [12,13]. Nonetheless, *Bmp7* knockout mice do not show any defect in limb polarity. However, a redundant function in anterior-posterior patterning with BMP2 remains a possibility. In addition to BMP2 and BMP7, a third member of this family that is closely related to BMP2, BMP4, is also expressed in the early limb bud. Like BMP7, it is expressed in both the anterior and posterior margins of the limb bud mesenchyme [4,14]; however, it does not appear to be induced by SHH signaling, nor does its expression change in SHH-deficient limb buds. Thus, BMP4 is not a candidate for a secondary signal downstream of SHH in early patterning. However, all three of these molecules, BMP2, BMP4, and BMP7, have been suggested to act in a second distinct phase of limb patterning, when digit identities are established downstream of earlier patterning events. In the vertebrate limb, each digit can be uniquely identified based on its size, length, number of phalanges, and location within the autopod. As a consequence of the initial establishment of anterior-posterior positioned information within the limb by SHH and/or BMP signaling, the interdigital mesenchyme of the hand plate becomes specified in a graded manner. Grafting and extirpation experiments have shown that it is this polarized interdigital tissue which directs digit morphology [15]. BMP2, BMP4, and BMP7 are all expressed in the interdigital mesenchyme [4,14], and experiments inhibiting BMP signaling in this tissue suggested that differential levels of BMPs in the interdigital mesenchyme might be the relevant factor directing digit morphology [15]. As noted above, BMP7-deficient embryos produce mostly normal limbs, and mice harboring a conditional deletion of *Bmp4* in the limb bud mesenchyme also do not show evidence of anterior digit transformation [16], as would have been predicted if BMP4 acted as an interdigital regulator of digit morphology. In the light of these observations, it would seem unlikely that quantitative differences in levels of interdigital BMP signal are responsible for establishing digit identity, although redundancy between BMP2, BMP4, and BMP7 in this role remains a possibility.

A third aspect of limb patterning in which BMP2, BMP4, and BMP7 have been implicated is in the apoptotic death of the interdigital tissue. For example, ectopic application of BMP antagonists demonstrates that BMP signaling is necessary for this process, and in its absence, webbing occurs [17–19]).

BMP activity also plays an indirect role in limb patterning as a key component of a feedback loop between SHH production in the posterior limb bud and fibroblast growth factor production in the overlying apical ectodermal ridge (AER). SHH activity leads to the upregulation of the BMP antagonist Gremlin in the mesenchyme [20,21]. This, in turn, prevents BMPs from downregulating fibroblast growth factor (FGF) production in the AER [17,22] and maintains the integrity of the AER itself [23]. FGF signaling feeds back to maintain SHH production in the posterior mesenchyme [8,24]. Thus, conditional removal of BMP4 activity from the posterior limb mesenchyme results in a persistence of the AER, expanded SHH signaling, and consequent preaxial and postaxial polydactyly [16].

At later stages of limb development, BMP activity is also believed to play critical roles in skeletogenesis. Activation and dominant-negative experiments in the chick have shown that signaling through the BMPR-IB receptor is necessary and sufficient for cartilage condensation in chick [25]. Likewise, conditional knockout of *BmpR1A* and *BmpR1B* blocks all chondrogenic differentiation in the mouse limb bud [26]. Similarly, when *noggin*, a potent BMP inhibitor, is introduced in early stage chick limb buds prior to skeletogenesis, mesenchymal condensation does not take place [27,28]. Taken together, these studies strongly implicate BMP signaling as necessary for mesenchymal condensations and the initiation of chondrogenesis. However, the phenotypes in these experiments are severe, causing either global chondrogenesis or absence of chondrogenesis, in the gain- and loss-of-function experiments, respectively. These severe defects during the early stages of skeletogenesis preclude investigation of any specific roles for BMPs in later events in cartilage differentiation, bone formation, and bone metabolism [29]. Conversely, removal of individual ligands such as BMP4 [16] or BMP7 [12,13] has not displayed defects in skeletal differentiation, presumably due to functional redundancy.

To gain further insights into the potential roles of BMP signaling in vertebrate limb patterning and skeletogenesis, we have produced mice simultaneously lacking BMP2 and BMP4 activity or BMP2 and BMP7 activity in the limb mesechyme during limb development. Since *Bmp2* and *Bmp4* mutants are both lethal early in embryogenesis [30,31], we used conditional alleles of both these genes. The conditional alleles were deleted early in limb development through the action of a *cre*-recombinase transgene expressed under the control of the *Prx1* enhancer [32]. Neither limbs deficient in BMP2 and BMP7 nor those deficient in BMP2 and BMP4 had defects suggestive of loss of anterior-posterior patterning information, arguing against these factors acting as secondary signals downstream of SHH or acting as interdigital determinants of digit identity. The *Bmp2*/*Bmp4* double mutants did display a loss of posterior digits with a broad posterior hand plate, suggestive of a block in the chondrogenic condensation of the posterior digit rays, rather than a change in anterior-posterior digit identity. At later stages, the combined activity of neither BMP2 and BMP4 nor of BMP2 and BMP7 is required for chondrogenesis. Osteogenesis is also initiated properly in the absence of these pairs of factors. However, at later stages, the combined loss of BMP2 and BMP4 evidently becomes limiting, and osteogenesis ceases. As all prior reported manipulations of the BMP pathway during skeletal development either result in a block at the first stage of chondrogenesis or allow complete skeletal formation, this is the first demonstration of a requirement for BMP activity in osteogenic differentiation.

## Results

**Figure 1:**
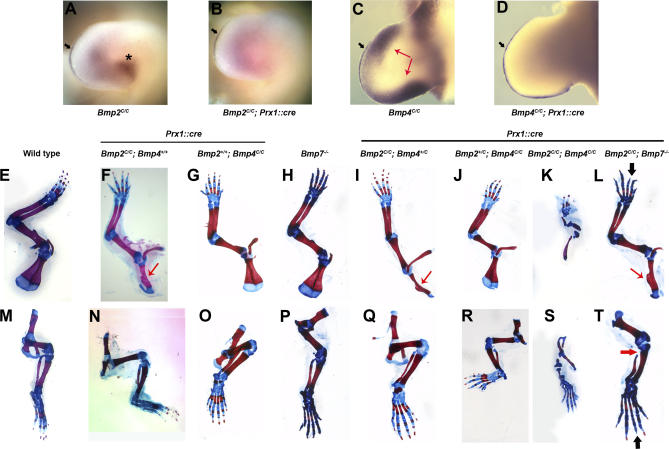
An Allelic/Nonallelic Series of BMP-Deficient Limbs (A–D) *Prx1::cre* efficiently recombines *Bmp2* and *Bmp4* conditional alleles in the limbs. *Bmp2* (A and B) and *Bmp4* (C and D) whole mount mRNA in situ hybridization in the limb. Wild-type (A) and *Bmp2^C/C^*; *Prx1::cre* (B) are forelimbs from E10.5 mouse embryos. Mesenchymal expression [asterisk (A)] of *Bmp2* is abolished in *Bmp2^C/C^*; *Prx1::cre* embryo while the AER expression [black arrow (A and B)] of *Bmp2* persists. Note that pink staining in the central region of the limb bud in (B) is nonspecific background. Wild-type (C) and *Bmp4^C/C^*; *Prx1::cre* (D) are forelimbs from E10.5 mouse embryos. Mesenchymal expression [red arrow (C)] of *Bmp4* is abolished in *Bmp4^C/C^*; *Prx1::cre* embryo while the AER expression [black arrow (C and D)] of *Bmp4* persists. (E–T) Depletion of BMP2 and BMP4 together causes severe limb skeletal defects. (E–T) Whole mount skeletons from newborn animals stained with Alcian blue and Alizarin red. (E–L) Forelimbs, (M–T) hindlimbs. (E and M) Wild-type, (F and N) *Bmp2^C/C^*; *Prx1::cre*, (G and O) *Bmp4^C/C^*; *Prx1::cre*, (H and P) *Bmp7* ^−*/*−^, (I and Q) *Bmp2^C/C^*; *Bmp4^+/C^*; *Prx1::cre*, (J and R) *Bmp2^+/C^*; *Bmp4^C/C^*; *Prx1::cre*, (K and S) *Bmp2^C/C^*; *Bmp4^C/C^*; *Prx1::cre,* (L and T) *Bmp2^C/C^*, *Bmp7* ^−*/*−^; *Prx1::cre.* Thin red arrow in (F), (I), and (L), defective scapula; thick red arrow in (T), failure of fibula to articulate with knee, and thick black arrow in (L) and (T), missing phalanx in digit III.

To investigate the roles of BMP signaling at various stages of limb patterning and skeletogenesis, we constructed a series of mice deficient singly or in combination in the ability to produce BMP2, BMP4, and BMP7. BMP7-deficient mice (kindly provided by Dr. Liz Robertson) survive until birth. However, BMP2 and BMP4 are both required for viability early in embryonic development [30,31]. We therefore constructed a conditional allele of *Bmp2*, introducing loxP sites flanking exon 3. We obtained a conditional allele of *Bmp4*, in which exon 4 is flanked by loxP sites, from Dr. Holger Kulessa and Dr. Brigid Hogan [16]. Both of these alleles would be expected to result in null alleles following recombination (see Materials and Methods for details). To conditionally inactivate *Bmp2* and *Bmp4* in the limb, we used a well-characterized transgene in which *cre*-recombinase is expressed under the control of the *Prx1* limb enhancer [32]. This transgene expresses *cre* very early in limb development, resulting in complete recombination of floxed alleles at early limb bud stages. We verified the ability of *Prx1::cre* to recombine the conditional *Bmp2* and *Bmp4* alleles at earliest limb bud stages using in situ hybridization. *Bmp2* is first expressed in the limb mesenchyme at embryonic day (E)10.5 in the mouse (Figure 1A, asterisk). By the time its expression is first detectable, the floxed *Bmp2* allele appears to be completely recombined in the limb mesenchyme, as whole mount in situ hybridization does not detect any mesenchymal *Bmp2* transcription (Figure 1B). At E10.5 *Bmp4* is expressed in the mouse limb mesenchyme in two stripes at the anterior and posterior margins (Figure 1C, red arrows). These expression domains are completely lost by E10.5 in the presence of the *Prx1::cre* transgene (Figure 1D). *Bmp2* and *Bmp4* are also expressed in the AER, where *Prx1::cre* is inactive, and these domains of expression are not affected (Figure 1A–1D, black arrows).

### An Allelic/Nonallelic Series of BMP-Deficient Limbs

**Figure 2:**
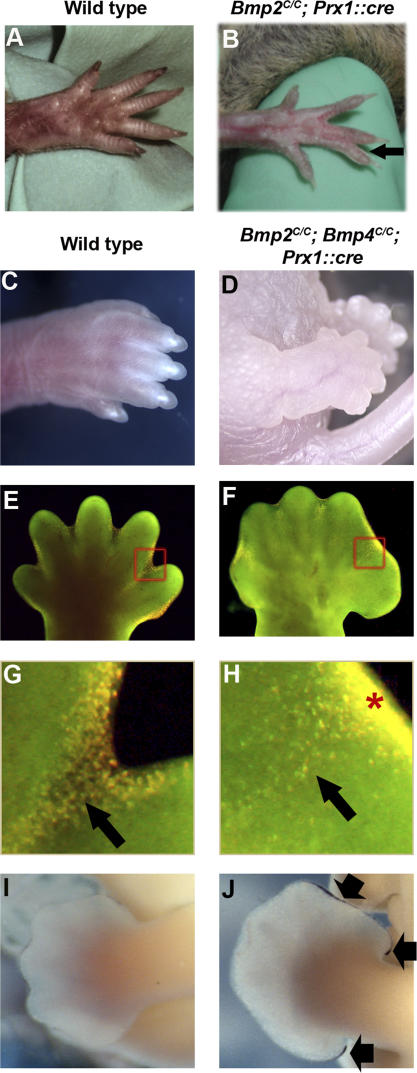
Depletion of BMP Signaling Causes Interdigital Syndactyly (A–D) Forelimb of adult wild-type (A) and *Bmp2^C/C^*; *Prx1::cre* mouse (B) and hindlimbs of newborn wild-type (C) mouse and newborn *Bmp2^C/C^*; *Bmp4^C/C^*; *Prx1::cre* (D) mouse. The black arrow in (B) shows soft tissue syndactyly in *Bmp2^C/C^*; *Prx1::cre* mouse. (E and F) Wild-type and *Bmp2^C/C^*; *Bmp4^C/C^*; *Prx1::cre*, respectively, showing acridine orange–stained hindlimbs of E15.5 mouse embryos. Acridine orange stain is in yellow. (G and H) Enlarged views of selected regions from (E) and (F), respectively. Black arrow in (G) and (H) show acridine orange–stained apoptotic cells in the interdigital mesenchyme, and asterisk in (H) shows the remnant of the AER. (I and J) *Fgf8* mRNA expression in the hindlimbs of E13.5 wild-type (I) and *Bmp2^C/C^*; *Bmp4^C/C^*; *Prx1::cre* (J) embryos. The thick black arrows in (J) show Fgf8 mRNA expression.

Mice were generated with limbs deficient in BMP2, BMP4, or BMP7, both BMP2 and BMP4, or both BMP2 and BMP7. To obtain an initial indication of the range of phenotypes produced in these animals, we first examined the limb skeletons of newborn animals. BMP2-deficient limbs appeared remarkably normal, both in skeletal pattern and in gross aspects of chondrogenesis and osteogenesis indicated by Alcian blue/Alizarin red staining. The one notable defect in the appendicular skeleton of these animals is a characteristic malformation of the scapula (Figure 1, red arrow). BMP2-deficient limbs also exhibit 3/4 soft-tissue syndactyly with variable penetrance (Figure 2).

Mice with limbs deficient in BMP4 activity and the limbs of *Bmp7* mutant mice have both been previously described. As reported [16], in the absence of BMP4 activity, limbs display a variable penetrance of preaxial and postaxial polydactyly, but otherwise, normal digit patterns and apparently normal skeletal differentiation (Figure 1G and 1O) take place. As previously described, mice homozygous for a null mutation in *Bmp7* [12] have no defects in the formation of the normal appendicular skeletal elements (Figure 1H and 1P). We do occasionally observe preaxial polydactyly in these mutants.

Compound heterozygous mice, with one functional copy each of *Bmp2* and *Bmp4* in the limb (*Bmp2^+/C^*; *Bmp4^+/C^*; *Prx1::cre*), show no effect on either limb patterning or skeletogenesis (unpublished data). Similarly, limb skeletons of mice in which both copies of the *Bmp4* gene and one copy of the *Bmp2* gene have been conditionally removed in the limb (*Bmp2^+/C^*; *Bmp4^C/C^*; *Prx1::cre)* are phenotypically normal other than exhibiting the variable penetrance preaxial and postaxial polydactyly seen in mice deficient in BMP4 activity alone (Figure 1J and 1R). In contrast, mice in which both copies of *Bmp2* and one copy of *Bmp4* had been removed (*Bmp2^C/C^*; *Bmp4^+/C^*; *Prx1::cre*) showed more severe skeletal defects, including significantly thinner skeletal elements. However, the digit patterns of those animals are completely normal (Figure 1I and 1Q). Animals in which both copies of *Bmp2* and *Bmp4* were removed (*Bmp2^C/C^*; *Bmp4^C/C^*; *Prx1::cre*) had extremely malformed limbs (Figure 1K and 1S). They displayed severely short and malformed stylopods; one of the zeugopod elements was almost always missing, and the remaining one was so deformed that it was difficult to identify the element correctly. Moreover, the joint articulations are defective such that zeugopod and stylopod elements are fused (see below and Figure S1A–S1C). Interestingly, the autopods are less affected than the proximal elements. Nonetheless, the autopod elements are significantly reduced in size, and strikingly the two posterior-most digits are missing in the forelimbs of these animals.

Simultaneous removal of BMP2 and BMP7 had far less of an effect than did removal of BMP2 and BMP4 activity. Compound heterozygous removal of BMP2 and BMP7 (*Bmp2^+/C^, Bmp7^+/^* ^−^; *Prx1::cre*) and heterozygous removal of BMP2 with complete removal of BMP7 (*Bmp2^+/C^*, *Bmp7* ^−*/*−^; *Prx1::cre*) were both completely wild-type in skeletal pattern and differentiation (unpublished data). Mice homozygous for removal of *Bmp2* and heterozygous for *Bmp7* (*Bmp2^C/C^*, *Bmp7^+/^* ^−^; *Prx1::cre*) displayed the same subtle scapular defect seen in *Bmp2^C/C^*; *Prx1::cre* mutants alone (unpublished data). In limbs developing in the complete absence of both BMP7 and BMP2 activity (*Bmp2^C/C^*, *Bmp7* ^−*/*−^; *Prx1::cre*), the last phalanx was missing from digit III in the forelimb, and these limbs displayed the same phenotype in the hindlimb with variable penetrance (Figure 1L and 1T, black arrow). Additionally, the fibulae of these hindlimbs are malformed and do not articulate with the femur at the knee (Figure 1T, thick red arrow). They also display the same scapular defects seen in *Bmp2^C/C^*; *Prx1::cre* mice, and the overall size of the appendicular skeleton is slightly diminished. However, skeletal differentiation appears normal in the limbs of these animals.

### Soft Tissue Syndactyly Observed in Limbs of Mice Devoid of BMP2 and BMP4 Activity

As noted above, *Bmp2*-deficient animals display a variably penetrant 3/4 soft tissue syndactyly phenotype (Figure 2B). In contrast, *Bmp4*-deficient mice show no evidence of syndactyly (unpublished data). However, one of the striking defects observed in the *Bmp2^C/C^*; *Bmp4^C/C^*; *Prx1::cre* mice is that in the newborn animals, the digits of both forelimb (unpublished data) and hindlimb show complete syndactyly (Figure 2D; compare to wild-type, Figure 2C). To determine the origin of this defect, we examined embryonic limbs at E15.5, the stage when interdigital separation is normally taking place (Figure 2E). In *Bmp2^C/C^*; *Bmp4^C/C^*; *Prx1::cre* limbs, only the very distal tips of each digit were separated, with the autopod adopting the shape of a notched pallet (Figure 2F). In wild-type development, the individual digits become free from one another through a process of interdigital apoptosis, observable by staining with acridine orange (Figure 2E and 2G, arrow). Interdigital apoptosis is dramatically reduced in the *Bmp2^C/C^*; *Bmp4^C/C^*; *Prx1::cre* limbs (Figure 2F and 2H, arrow). (Note that the strong acridine orange staining in Figure 2H is not in the interdigital mesenchyme but rather is in the remnant of the cells of the AER [red asterisk].)

Another region of very prominent apoptosis in the wild-type limb bud is within the AER. The AER is a specialized ridge of ectoderm running along the anterior-posterior axis at the distal margin of the early limb bud. One of the phenotypes characterizing the *Bmp2^C/C^*; *Bmp4^C/C^*; *Prx1::cre* mutant is expansion of the AER (see below). Moreover, in the *Bmp2^C/C^*; *Bmp4^C/C^*; *Prx1::cre* animals, the AER is maintained as late as E15.5, long after it has started to degrade in wild-type limbs. This can be seen morphologically (unpublished data) or by staining for expression of AER-specific marker such as *Fgf8* (Figure 2I and 2J). The AER displays intense staining with acridine orange, TUNEL, or other markers for apoptosis at every stage when it is present, reflecting the rapid turnover of cells in this structure [33–35]. The *Bmp2^C/C^*; *Bmp4^C/C^*; *Prx1::cre* mutant limbs at E15.5 show strong acridine orange staining in the remnants of AER (Figure 2H, asterisk), in domains of the distal tip where the AER remains the longest (compare staining in Figure 2F and 2J). No significant difference was observed in the extent of apoptosis in the cells of the AER in earlier stage *Bmp2^C/C^*; *Bmp4^C/C^*; *Prx1::cre* mutant embryonic limbs (Figure S1D–S1G).

### Patterning Defects in Limb Developing with Reduced Levels of BMP Signaling

**Figure 3:**
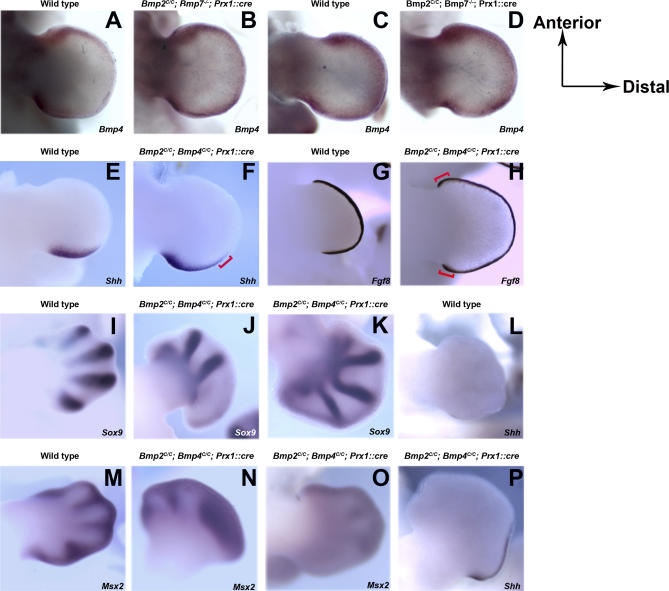
Patterning Defects in Limbs Deficient of Different Combinations of BMP Molecules (A–D) *Bmp4* expression in limb buds from E11.5 wild-type (A and C) and *Bmp2^C/C^*, *Bmp7* ^−*/*−^; *Prx1::cre* (B and D) mouse embryos. (A and B) Forelimbs, (C and D) hindlimbs. (E–H) *Shh* (E and F) and *Fgf8* (G and H) expression in E11.5 forelimbs and hindlimbs, respectively. (E and G) Wild-type embryos, (F and H) *Bmp2^C/C^*; *Bmp4^C/C^*; *Prx1::cre* embryos. (I–P) *Sox9* (I–K) and *Msx2* (M–O) expression in E12.5 wild-type (I and M) and *Bmp2^C/C^*; *Bmp4^C/C^*; *Prx1::cre* mouse embryonic forelimbs (J and N) and hindlimbs (K and O). (L and P) *Shh* expression in E12.5 wild-type (L) and *Bmp2^C/C^*; *Bmp4^C/C^*; *Prx1::cre* (P) embryonic hindlimbs. The red brackets in (F) and (H) show the broadened domains of expressions of *Shh* and *Fgf8*, respectively.

BMP signaling has been proposed to play several distinct roles in limb patterning. The first of these is in establishing differences along the anterior-posterior axis of the limb downstream of *sonic hedgehog (Shh).* SHH signaling itself extends over a long range in the developing limb bud, based on both activation of target genes [36] and protein distribution [37]. Nonetheless, some models have suggested that the patterning effect of this morphogenic signal is mediated entirely [10] or in part [11] by BMP2 and BMP7 produced as secondary signals in response to SHH. We have removed both BMP2 and BMP7 activities in the early limb bud (Figure 1), and moreover, there is no apparent upregulation of BMP4 expression in the posterior of these limbs (Figure 3A–3D). The skeletal phenotypes of mouse limbs formed in the absence of BMP2, BMP7, and both BMP2 and BMP7 are illuminating, as none of these exhibit significant digit pattern defects (Figure 1). While a distal phalanx is missing in the middle digits of the double mutants (*Bmp2^C/C^*, *Bmp7* ^−*/*−^; *Prx1::cre*), this is not a phenotype consistent with a decrease or loss of a posteriorly derived polarizing activity. Indeed, the terminal phalanx forms at the distal tip of each digit by a distinct mechanism, not dependent upon anterior-posterior patterning [38]. These data strongly indicate that BMP2 and BMP7 are not required secondary signals for SHH-mediated polarization of the digits along the anterior-posterior axis.

The lack of transformations in digit identity in the limb skeletons formed in the absence of BMP2 and BMP7 activity also argues against BMP signaling being the critical instructive signal from the interdigital mesenchyme to the forming digits. While a distal phalanx was lost from the middle digit of the forelimbs of these animals, these digits do not otherwise have a morphology consistent with their being transformed anteriorly into digit 1 (thumb). Indeed, in the hindlimbs, where the penetrance of the phenotype is variable, the mutant middle digits are otherwise indistinguishable from wild-type, other than the presence or absence of this variable skeletal element.

In contrast to the proposed roles for BMPs in anterior-posterior digit patterning, we obtained further support for the well-accepted idea that a threshold level of BMP signaling is necessary interdigitally for programmed cell death to occur. We observed syndactyly with variable penetrance in limbs developing without BMP2 activity (Figure 2B), and nearly complete soft tissue syndactyly of all digits in limbs deficient in both BMP2 and BMP4 (compare Figure 2C and Figure 2D).

The one dramatic patterning defect we observed in our BMP deficiency series was the loss of posterior digits in forelimbs deficient in both BMP2 and BMP4 (Figure 1K), also observed in earlier limb buds stained to show no expression of the condensation marker *Sox9* (Figure 3). To understand this phenotype, we analyzed limb buds at various stages using a series of molecular markers. By E11.5, the *Bmp2^C/C^*; *Bmp4^C/C^*; *Prx1::cre* limb buds are noticeably broader, expanded in the posterior relative to wild-type. This correlates with an anterior expansion in *Shh* expression (Figure 3E and 3F, red bracket) and a concomitant expansion of the AER as visualized by *Fgf8* expression (Figure 3G and 3H, red bracket). The expansion in *Shh* expression can first be detected at E10.5 and persists at least until E12.5, well after the time normal *Shh* expression disappears at E11.75 (Figure 3L and 3P). The expansion of the AER in the absence of BMP signaling may be understood on the basis of the feedback loop between the zone of polarizing activity (ZPA) and the AER. Expression of several *Fgf* genes [20,21] and maintenance of the AER itself [23] normally depend on BMP antagonism induced by SHH. Thus, a decrease in BMP signaling expands and maintains the AER. Because *Shh* expression is, reciprocally, supported by FGF signaling from the AER [8,24], this, in turn, feeds back on the ZPA, expanding *Shh* expression. While this explains the broader hand plate of the BMP2, BMP4–deficient limb buds, it is somewhat paradoxical in terms of the skeletal phenotype, as a broadened autopod would normally be expected to result in the formation of extra digits (polydactyly) (e.g., Figure 1G and 1O of this paper and [16]), not the loss of digits, as observed in the double mutant forelimbs.

To examine the loss of digits more closely, BMP2, BMP4–deficient limb buds were examined during cartilage condensation phase of the digit rays at E12.5. In wild-type limb buds the condensation of all five digits can be visualized at this stage by in situ hybridization with a probe directed against *Sox9*, the earliest known chondrogenic marker (Figure 3I). However, in *Bmp2^C/C^*; *Bmp4^C/C^*; *Prx1::cre* limb buds at E12.5, *Sox9* only detects primordia of the anterior three digits of the forelimb, indicating a failure in the formation of posterior digits at this earliest stage (Figure 3J).

Genes such as Msx2 (Figure 3N) continue to be expressed throughout the posterior forelimb mesenchyme, indicating that the posterior forelimb tissue is viable at E12.5 in the *Bmp2^C/C^*; *Bmp4^C/C^*; *Prx1::cre* animals. *Msx2* is normally expressed in the non-chondrogenic interdigital mesenchyme at this stage (Figure 3M), a continuation of an earlier expression domain throughout the distal mesenchyme. This expression of *Msx2* in the posterior of the BMP2, BMP4–deficient forelimbs reflects a failure of chondrogenesis in this region. The loss of the posterior forelimb digits in these mice thus does not appear to represent a decrease in polarizing activity leading to a change in digit identity rather a consequence of the overall level of BMP signaling falling below a threshold for initiating chondrogenesis in the posterior of these forelimbs. The posterior of the hindlimb in the mutant, in contrast, appears to remain above this threshold, and more than five digit condensations form in the hindlimb bud (Figures 3K and 1S) and no ectopic expression of *Msx2* is observed in the posterior (Figure 3O).

### Skeletal Differentiation in Limbs Developing with Reduced Levels of BMP Signaling

BMPs were first discovered in the context of their skeletogenic activity. We therefore wanted to determine whether the loss of the specific BMP molecules under investigation here would perturb the skeletal differentiation pathways. Accordingly, skeletal preparations from each of the various mutant alleles were subjected to histological and molecular analyses. The skeletal elements that formed in the absence of BMP2, BMP4, or BMP7 or BMP2 and BMP7 all underwent normal chondrogenesis and osteogenesis by a series of criteria. All showed normal staining with Alcian blue (for cartilage) and Alizarin red (for mineralized tissue, see Figure 1). Most were also analyzed in histological sections stained with toluidine blue and showed osteoid formation and bone matrix deposition (compare Figure S2A and S2E; and unpublished data). The skeletal elements in these various BMP-deficient animals contained normal prehypertrophic chondrocytes expressing *type II collagen*, hypertrophic chondrocytes expressing *type X collagen*, as well as the osteoblasts expressing *type I collagen*, like their wild-type counterparts (Figure S2; compare Figure S2F–S2H with Figure S2B–S2D; and unpublished data). In contrast, however, limbs completely deficient in both BMP2 and BMP4 activity showed severe defects in skeletal differentiation.

### BMP2 and BMP4 Are Not Critical for Chondrogenic Differentiation

**Figure 4:**
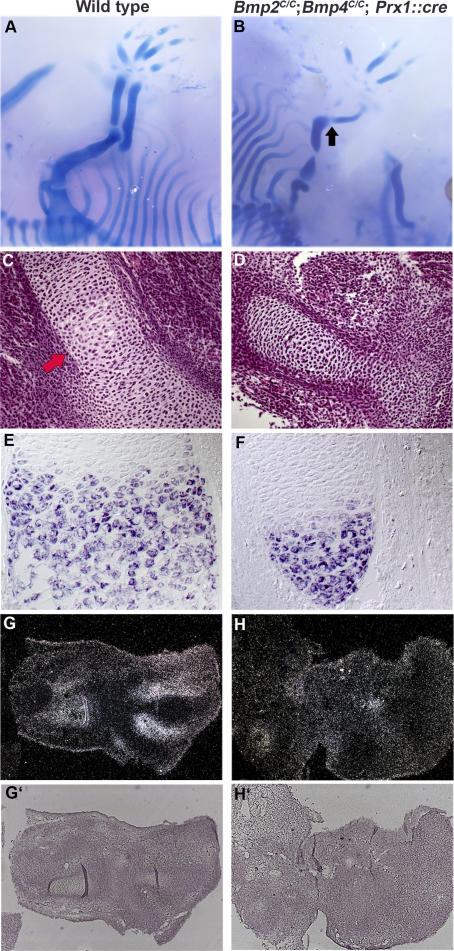
Chondrogenesis Starts and Proceeds Normally Even in the Absence of BMP2 and BMP4 (A and B) Whole mount skeletons from E13.5 embryos that are stained with alcian blue. (A) Wild-type embryo, (B) *Bmp2^C/C^*; *Bmp4^C/C^*; *Prx1::cre* embryo. Black arrow in (B) shows the fusion of the zeugopod and stylopod. (C and D) Hematoxylin and eosin–stained sagittal sections of humeri from E13.5 wild-type (C) and *Bmp2^C/C^*; *Bmp4^C/C^*; *Prx1::cre* (D) embryos. Thick red arrow in (C) shows the hypertrophic region. (E and F) Sagittal sections of wild-type (E) and *Bmp2^C/C^*; *Bmp4^C/C^*; *Prx1::cre* (F) humeri from E15.5 embryos were hybridized with digoxigenin-labeled antisense rioprobes for *ColX*. (G and H) Sagittal sections of forelimbs from E13.5 wild-type and BMP2, BMP4–deficient embryos, respectively, stained with radioactive riboprobes for *Bmp7* mRNA. (G′) and (H′) show the bright field views of (G) and (H), respectively.

As noted above, at E12.5, chondrogenic condensation of the digits could be visualized in *Bmp2^C/C^*; *Bmp4^C/C^*; *Prx1::cre* limb buds by in situ hybridization with a *Sox9* probe. Similar analysis at E10.5 and E11.5 demonstrated a relatively normal process of condensation of the proximal skeletal elements (Figure S1H–S1M), despite the absence of detectable *Bmp2* and *Bmp4* transcripts. As shown in Figure S1N and S1O, expression of *Col II*, a chondrocyte marker, is observed in the BMP2, BMP4–deficient cartilage (Figure S1O) as early as E12.5, similar to a wild-type control (Figure S1N). Similarly, at E13.5, the entire limb skeleton could be visualized undergoing chondrogenesis by Alcian blue staining, albeit in a defective pattern, in the BMP2, BMP4–deficient limb buds (Figure 4A and 4B and unpublished data).

As the cartilage elements grow, a wave of hypertrophic differentiation occurs from the center toward the distal ends (reviewed in [39–41]). Histological examination of the humeri at E13.5 showed that while chondrogenic differentiation had taken place in the BMP2, BMP4–deficient humerus, hypertrophic differentiation was delayed (Figure 4C and 4D). However, by E15.5, hypertrophy was evident in these elements as seen by *type X collagen* expression (Figure 4E and 4F). The hypertrophic cells in BMP2, BMP4–deficient limb also express late hypertrophic markers, such as *osteopontin* [42] (unpublished data).

To examine whether the successful execution of chondrogenesis in the BMP2, BMP4–deficient limbs is a result of compensatory upregulation of other ostegenic BMPs, we carried out in situ hybridization for *Bmp6* and *Bmp7* in these limbs. At E13.5, we observed no increase in expression of *Bmp6* (Figure S1P–S1S) or *Bmp7* (compare Figure 4H with Figure 4G) mRNA in the mutant tissues compared to their wild-type counterparts

### Osteogenesis Is Initiated Normally in BMP2, BMP4–Deficient Limb Buds

**Figure 5:**
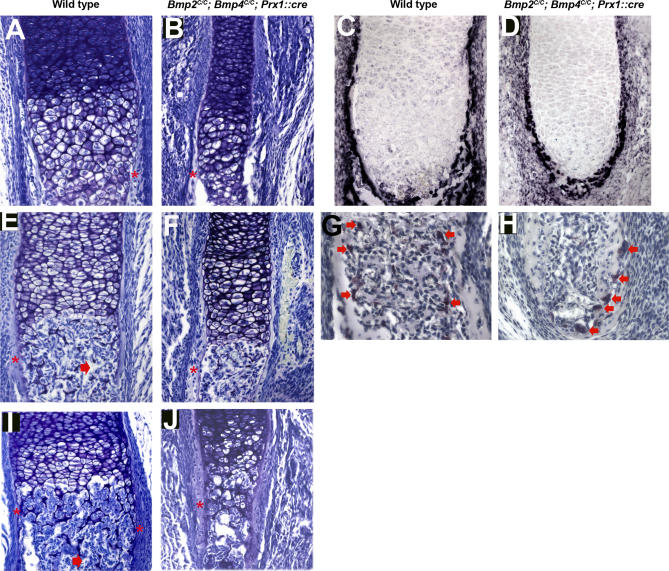
In the Absence of BMP2 and BMP4, Osteogenesis Begins During Early Embryonic Development (A–D) Sagittal sections of forelimb from E15.5 embryos. (A and B) Stained with toluidine blue. (A) Distal ulna from wild-type embryo, (B) distal ulna/radius from *Bmp2^C/C^*; *Bmp4^C/C^*; *Prx1::cre* embryo. Mineralized cartilage is shown by dark purple and osteoid is shown by light blue (red asterisk). (C and D) Humeri sections were hybridized with digoxigenin labeled riboprobe for *ColI*. (C) Section of wild-type humerus from E15.5 embryo, (D) from *Bmp2^C/C^*; *Bmp4^C/C^*; *Prx1::cre* E15.5 embryo. (E–H) Sagittal sections of zeugopod from E17.5 embryos. (E and F) Stained with toluidine blue while G and H are stained for TRAP (brown stain, red arrow). (E and G) E17.5 proximal radius from wild-type embryo, (F and H) E17.5 proximal ulna/radius from *Bmp2^C/C^*; *Bmp4^C/C^*; *Prx1::cre* embryo. Although vascularization occurs in the absence of BMP2 and BMP4, few osteoclasts are observed in the mineralized cartilage in *Bmp2^C/C^*; *Bmp4^C/C^*; *Prx1::cre* mice. Please note all the panels other than (G) and (H) are photographed at ×20, while (G) and (H) were photographed at ×40. (I and J) Sections of newborn proximal radius (I) from control animal and newborn proximal ulna/radius (J) from *Bmp2^C/C^*; *Bmp4^C/C^*; *Prx1::cre* animal were stained with toluidine blue. Red arrow shows the bone marrow cavity in (E) and (I). Note that bone collar (red *) forms at the right time and place in the absence of BMP2 and BMP4. Also note that since *Bmp2^C/C^*; *Bmp4^C/C^*; *Prx1::cre* mice occasionally have only one bone in zeugopod and the elbow joint is occasionally fused, it is difficult to distinguish between the ulna and the radius.

**Figure 6:**
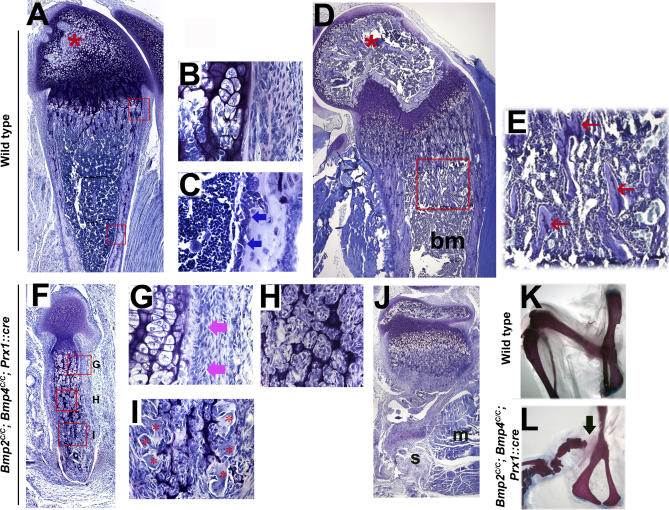
Defects in Bone Formation in the Absence of BMP2 and BMP4 Toluidine blue staining of sagittal sections of distal femurs. (A–E) Control femurs at 1 wk (A) and 3 wks (D) of age. Boxed areas in (A) are enlarged in (B) and (C). Boxed area in (D) is enlarged in (E). Blue arrows in (C) point to osteoblast cells lining the surface of cortical bone. Pink arrows in (G) show similar cells to those in (C) in *Bmp2^C/C^*; *Bmp4^C/C^*; *Prx1::cre* femurs. Red star in (A) and (D) marks the secondary ossification center. Pale blue–stained tissue in (E), marked by red arrows, is trabecular bone. bm, marks the bone marrow cavity in (D). (F–J) *Bmp2^C/C^*; *Bmp4^C/C^*; *Prx1::cre* femurs at 1 wk (F) and 3 wks (J) of age. Boxed areas in (F) are enlarged in (G), (H), and (I). (A), (D), (F), and (J) are shown with the same magnification. Note that there are defects in bone formation but no defect in osteoclast mediated bone resorption. Mineralized tissue in the mid shaft of femur is almost resorbed at 3 wks. (H) Fibroblast-like cells present in the bone shaft of *Bmp2^C/C^*; *Bmp4^C/C^*; *Prx1::cre* mouse. Red star in (I) shows the osteoclasts invading the *Bmp2^C/C^*; *Bmp4^C/C^*; *Prx1::cre* femurs at 1 wk of age. s and m mark soft tissues and muscle in (J), respectively. (K and L) Three-wk-old wild-type (K) and *Bmp2^C/C^*; *Bmp4^C/C^*; *Prx1::cre* (L) hindlimb skeletons stained with Alcian blue and Alizarin red. The black arrow shows the missing part of proximal femur.

Bone formation first occurs at the interface between the late hypertrophic chondrocytes and the surrounding perichondrium, a region identified as the bone collar. We examined whether the osteogenesis program is initiated in the BMP2, BMP4–deficient limbs using several histological and molecular criteria, as described below (Figure 5). Adjacent to the hypertrophic cells, a bone collar is laid down normally in the absence of BMP2 and BMP4 (Figure 5A and 5B, red star). As can be seen in sagittal sections through an E15.5 humerus, the cells embedded in the mineralized matrix express the osteoblast marker *Col I* as normal (Figure 5C and 5D). By E17.5, the mineralized cartilage that makes up the late hypertrophic zone of the growth plate normally undergoes removal by osteoclasts (Figure 5G, brown-stained cells, red arrows), resulting in the formation of a bone marrow cavity (red arrow in Figure 5E and 5I, see also Figure 6). Osteoprogenitor cells from bone collar region now populate this site and deposit osteoid, forming the trabecular bone in the bone marrow cavity (Figure 6E, red arrow) and cortical bone at birth (Figure 5I, asterisk). In contrast, in E17.5 limbs deficient in BMP2 and BMP4, there is an overall delay in the normal endochondral process (Figure 5F). While vascularization occurs in *Bmp2^C/C^*; *Bmp4^C/C^*; *Prx1::cre* forelimbs, few osteoclasts are recruited to the mineralized cartilage (compare Figure 5G and Figure 5H, red arrows), resulting in a delay in bone marrow cavity formation in the absence of BMP2 and BMP4. In fact, bone marrow formation and trabecular bone formation have not yet started at birth in the absence of BMP2 and BMP4 (Figure 5J), and the morphology of *Bmp2^C/C^*; *Bmp4^C/C^*; *Prx1::cre* bones at birth is most similar to control embryos at E17.5 (Figure 5E). To differentiate between a simple delay in bone formation in the absence of BMP2 and BMP4 and the inability to form bone without these BMPs, we performed histological analyses in *Bmp2^C/C^*; *Bmp4^C/C^*; *Prx1::cre* mice up to 3 wks of age. As shown in Figure 6A through 6E, in the control mice, a bone collar is found adjacent to the zone of late hypertrophic chondrocytes at 1 wk (Figure 6A and 6B). Many osteoblasts line the newly formed cortical bone in the diaphyseal region (Figure 6C, blue arrow), and formation of a secondary ossification center has begun (Figure 6A, red star). At 3 wks of age, formation of the secondary ossification center is complete (Figure 6D, red star), along with robust bone marrow formation (Figure 6D, bm) and deposition of trabecular bone (Figure 6E, pale blue tissue marked by red arrow). In stark contrast, bone formation is not observed in the absence of BMP2 and BMP4 at 1 or 3 wks after birth (Figure 6F–6J). The appearance of the skeletal elements in the double mutants remains similar to E17.5 structures seen in wild-type limbs (Figure 5E), and although there is a bone collar at the site of hypertrophic cartilage (Figure 6G), no bone marrow cavity, trabecular bone, or cortical bone is present (Figure 6F). Many osteoclasts have been recruited to the remaining mineralized cartilage and are actively resorbing this tissue (red asterisks, Figure 6I), so that by 3 wks, all the mineralized cartilage in the diaphyseal region has disappeared, leaving a void where bone formation should have occurred (Figure 6J, also see Figures 6L and S3). This void is eventually invaded by soft tissues and the adjacent muscle (marked s and m, respectively, in Figure 6J). The resorption of bone at 3 wks of age is clearly visible in the proximal femur of mutant animals where a portion of that bone is missing (Figure 6L, black arrow). The inability to complete osteoblast differentiation in the *Bmp2^C/C^*; *Bmp4^C/C^*; *Prx1::cre* mice results in strikingly defective limb morphology at 1 wk of age (compare Figure S3G and S3H).

**Figure 7:**
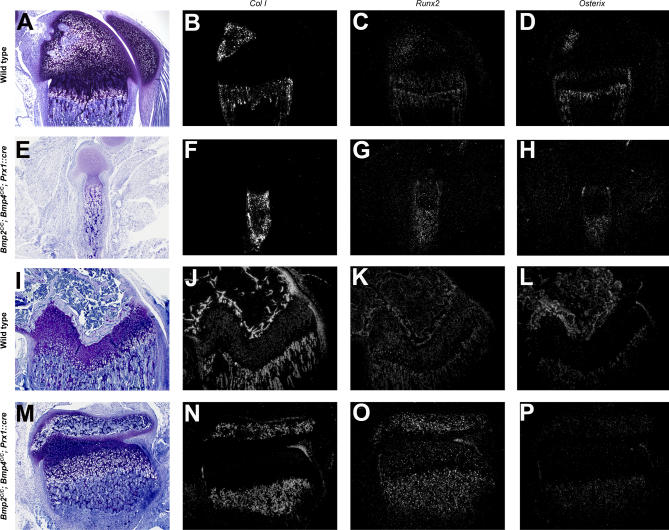
Osteoblast Maturation Is Inhibited in the Absence of BMP2 and BMP4 (A–D) Distal femurs from control mice at 1 wk of age. (E–H) Distal femurs from *Bmp2^C/C^*; *Bmp4^C/C^*; *Prx1::cre* mice at 1 wk of age. (I–L) Distal femurs from control mice at 3 wks of age. (M–P) Distal femurs from *Bmp2^C/C^*; *Bmp4^C/C^*; *Prx1::cre* mice at 3 wks of age. In situ hybridization of early osteoblast differentiation-related marker genes *Col I* (B, F, J, and N), *runx2* (C, G, K, and O) and *osterix* (D, H, L, and P).

To determine if any osteoblast differentiation occurs in *Bmp2^C/C^*; *Bmp4^C/C^*; *Prx1::cre* forelimbs, we examined osteoblast-related gene expression (Figure 7). In the control mice, osteoblasts lining the surface of trabecular and cortical bone express *type I collagen*, *runx2*, and *osterix* genes at both 1 wk and 3 wks after birth (Figure 7A–7D and 7I–7L). In the absence of BMP2 and BMP4, cells which are fibroblastic in appearance (Figure 6H) are found adjacent to the mineralized cartilage surfaces. These cells express amounts of *type I collagen* and *runx2* comparable to control osteoblasts, but their expression of osterix, while noticeable at 1 wk, becomes almost undetectable at 3 wks (Figure 7E–7H and 7M–7P). These results allow us to conclude that the fibroblast-like cells resident in the bone shaft (Figure 6H) may be osteoprogenitor cells unable to differentiate into mature osteoblasts in the absence of BMP2 and BMP4.

## Discussion

Using a genetic approach to remove activity of different *Bmp* genes during limb development, we have found that BMP2, BMP4, and BMP7 either individually or in combination are not required for specification of normal digit identities. However, BMP2 and BMP4 are required in concert to promote condensation of the posterior digit anlagen. We have also shown that chondrogenesis can be initiated and chondrogenic differentiation will take place even in the absence of both BMP2 and BMP4 or BMP2 and BMP7. On the other hand, BMP2 and BMP4 together are required for completion of osteogenesis.

### BMP Signaling and Pattern Formation

It has been proposed that BMP signaling may be involved in digit patterning as a secondary signal originating in the ZPA [7,11,43] or as a later mediator of interdigital patterning information [15]. In particular, BMP2, a secreted factor that is induced by SHH [8] in the posterior limb bud, has been suggested to act as a true morphogen secondary to SHH signaling to properly pattern the limb digits. However, inactivation of *Bmp2* during early limb bud development clearly does not cause any digit patterning defects. BMPs are known, in many cases, to function redundantly [44]. In considering potential secondary signals mediating ZPA activity, the most relevant family member to consider is BMP7 as, like BMP2 (and unlike BMP4), it is positively regulated by SHH signaling [7]. However, *Bmp7* expression does not overlap with *Bmp2* in the early mouse limb bud [45], and in any case, mice in which we conditionally knocked out *Bmp2* in the *Bmp7* ^−/−^ background do not show any obvious digit patterning defect other than the missing final phalanx in digit III. Although still poorly understood, the final phalanges form at the tip of every digit through a process distinct from the more proximal phalanges [38]. Our data implicate BMP signaling in this process.

At later stages, BMP signaling has been suggested to play a role in mediating the transfer of positional information from the interdigital mesenchyme to specify the identity of the adjacent digits [15]. Application of the BMP antagonist noggin to the interdigital tissue at the time of digit condensation results in loss of phalanges and a resultant pattern that can be interpreted as an anterior homeotic transformation in digit identity. However, increasing BMP concentrations interdigitally with exogenous protein does not result in the reciprocal posterior transformations, and the *noggin* experiments can also be interpreted simply as a block in the condensation of the primordia of distal phalanges when BMP activity is completely abolished [38]. Decreasing the level of interdigital BMP signaling, without abolishing it, provides an opportunity for differentiating between these models. If BMP signaling were only required as a permissive factor for chondrogenesis, partial reduction in the level of signaling might have no effect. However, if levels of BMP signaling were instructive in terms of digit identities, then a substantial decrease in the amount of BMP ligand present should cause transformations in digit identity. This is not what is observed, either in limbs deficient in both BMP2 and BMP4 or in those deficient in BMP2 and BMP7. It should be noted that the interdigital expression domains form several days after recombination of the floxed BMP alleles is complete. Moreover, there is no detectable compensatory upregulation of BMP7 in the interdigital mesenchyme of the BMP2, BMP4–deficient limbs (compare Figure 4G and Figure 4H). Thus, we conclude that BMP signaling is not likely to be responsible in a quantitative fashion for the instructive role of interdigital mesenchyme in establishing digit identities. However, in the *Bmp2^C/C^*; *Bmp4^C/C^*; *Prx1::cre* forelimbs, there is a complete loss of posterior digit ray condensations, presumably because the total level of BMP signaling falls below the threshold for initiation of condensation, as previously seen in cases where BMP antagonists have been used [27,28].

It is worth noting that the defects in chondrogenesis seen in the BMP2, BMP4–deficient limbs are much more severe proximally than distally. This is particularly significant because proximal elements condense and differentiate before distal ones. Thus, if the conditional alleles were partially recombined at early stages and only completely recombined at later stages, it is the distal elements, formed after greater recombination has taken place, that should be more severely affected. Since it is the proximal elements which are more severely affected, the explanation for the difference in severity must lie elsewhere. This is most likely explained by compensation in the distal limb bud by BMP2, BMP4, and BMP7 produced in the AER.

Compensation by BMPs in the AER also likely explains a difference seen between the *Bmp4^C/C^*; *Prx1::cre* mice in our experiments and a recently published analysis [16], which used the identical *Bmp4* conditional allele and *Prx1::cre* driver. The earlier report, like ours, described preaxial and postaxial polydactyly. However, they observed these phenotypes with less variability and, moreover, described multiple preaxial and postaxial ectopic digits, while we saw, at most, a single preaxial and postaxial digit. The difference is that the mice analyzed in the previous report had one null allele and one conditional allele, while ours had two conditional alleles. Their mice, therefore, lost a half-dose of BMP4 from the AER in addition to complete loss in the mesenchyme, while our conditional alleles were only recombined in the mesenchyme by the *Prx1::cre* driver. It is also possible that the difference in severity relates to the time required to recombine one as opposed to two floxed alleles; however, we do not favor this explanation as all detectable BMP4 expression is lost prior to any morphological differences between the mutant and wild-type limb buds. Finally, differences in genetic background could contribute to the differences as the *Prx1::cre* line is outbred.

A final aspect of patterning previously associated with BMP signaling is interdigital apoptosis. Consistent with this, we see some syndactyly in the BMP2-deficient limbs. The fact that this is variable, and limited to the 3/4 interdigit, implies that the threshold for induction of apoptosis is low enough that loss of BMP2, BMP4 or BMP7 or BMP2 and BMP7 still leaves sufficient BMP signaling for most interdigital cell death to occur normally. However, loss of both BMP2 and BMP4 results in complete soft tissue syndactyly, i.e., webbing between all the digits. Although BMP activity has been previously implicated in regulating interdigital apoptosis, this is the first genetic verification of their requirement for this process.

### BMP Signaling and Skeletal Development

As discussed above, our data indicate that threshold levels of BMP signaling are required for initiating chondrogenic condensation, consistent with prior results [26–28]. However, once chondrogenesis begins, cartilage differentiation can be sustained, albeit with some detectable delay, in the absence of BMP2 and BMP4 or in the absence of BMP2 and BMP7. Thus, the expression of either BMP5, BMP6, and BMP7 (in the absence of BMP2 and BMP4) or the expression of BMP4, BMP5, and BMP6 (in the absence of BMP2 and BMP7) is sufficient to support mesenchymal condensation and chondrocyte differentiation in the developing limb.

In one set of previous studies implicating BMP signaling in chondrogenesis, Yoon et al. [26] showed that if mice lose both *Alk3 (BmprIa)* and Alk6 *(BmpIb),* two of the three type I receptors used in BMP signal transduction, there is a dramatic decrease in the size of skeletal primordial due to a reduction of proliferation and increase in apoptosis. The size of the skeletal elements in *Bmp2^C/C^*; *Bmp4^C/C^*; *Prx1::cre* limbs are also decreased compared to those of control mice, although not to the extent seen in the double receptor mutant. We do not know if reduced size of the skeletal elements in our study is similarly due to reduced proliferation and increase in apoptosis, lack of replacement by bone, or a combination of these. It is possible that a triple mutant removing BMP2, BMP4, and BMP7 activities would have a similar phenotype as the *BmprIa*, *BmprIb* double mutant, although these could also be compensated from other BMPs. We do know, however, that osteogenesis is defective in these mice, although bone formation proceeds normally in siblings retaining one functional copy of either the *Bmp2* or *Bmp4* gene (Figure S4), as it does in mice completely deficient in BMP2 and BMP7 (Figures 1 and S2).

The phenotype we observe in limbs of *Bmp2^C/C^*; *Bmp4^C/C^*; *Prx1::cre* mice is similar to the phenotype reported for osterix knockout mice [46], which exhibited severe defects in osteoblastic differentiation. Since we observe fibroblastic cells adjacent to mineralized cartilage that have characteristics of osteoprogenitor cells but fail to express osterix in the absence of both BMP2 and BMP4, one important role of BMP2 and BMP4 during endochondral ossification may be to induce osterix gene expression in osteoprogenitors. Strikingly, however, we do observe osterix expression in the bone collar of E16.5 mice missing functional copies of both the *Bmp2* and *Bmp4* genes (K. Tsuji and A. Bandyopadhyay, unpublished data). This suggests that varying levels of expression from different BMP genes during preaxial and postaxial development result in fluctuations in total levels of BMP signaling, which at distinct times are above or below the threshold for supporting osteogenesis. While many osteogenic BMP molecules, apart from BMP2 and BMP4, such as BMP5, BMP6, and BMP7, are expressed during the endochondral process, our data suggest that these BMP molecules cannot compensate for the combined loss of BMP2 and BMP4 in bone formation. Recent biochemical and genetic experiments have suggested that individual BMPs have identical functions. BMP2, BMP4, BMP5, BMP6, and BMP7 can utilize the same type I (Alk2, Alk3, and Alk6) and type II receptors (BMP RII, ActRII, and ActRIIb) [47]. Once the complex between ligand and receptors is formed, BMP2, BMP4, BMP5, BMP6, and BMP7 direct the phosphorylation of the same set of BMP receptor–specific Smads (1, 5, or 8), and the signal that is transduced by each BMP appears to be identical in the skeletal target cells [48–51]. BMPs have also been shown to activate MAPK and AKT pathways and, based on current published information, each of the osteogenic BMPs appears to have the same capacity to activate these signaling pathways [52–54]. When this information is considered along with our observation that one allele of either *Bmp2* or *Bmp4* can rescue the osteoblast differentiation phenotype observed in *Bmp2^C/C^*; *Bmp4^C/C^*; *Prx1::cre* mice (see Figures 1I, 1J, 1Q, and 1R and S4), we favor the hypothesis that bone formation requires a threshold amount of BMP signaling which is not met when both BMP2 and BMP4 are completely absent. We cannot, however, exclude the possibility that BMP2 and BMP4 have distinct functions in osteoblastogenesis. Our analysis of skeletogenesis indicates that BMP2 and BMP4 are prerequisite for osteoblastogenesis while less important for chondrogenesis. These studies provide the first evidence linking BMP2 and BMP4 with bone formation in an in vivo setting where the preceding events of chondrogenesis are not compromised.

## Materials and Methods

### Reagents.

Xylene, formamide, and acetic anhydride were purchased from Fisher Scientific (http://www.fishersci.com). Alizarin red S, toluidine blue, paraformaldehyde, polyvinylpyrrolidone, proteinase K, RNase A, tRNA, and dithiothreitol were purchased from Sigma (http://www.sigmaaldrich.com). Alcian blue 8GX was purchased from Electron Microscopy Science (http://www.emsdiasum.com). Dextran sulfate was purchased from Pharmacia (http://www.pharmacia.com).

### Generation of *Bmp2* and *Bmp4* conditional null allele.

Mice carrying floxed *Bmp2* allele were available through a material transfer agreement between Harvard University and Wyeth Pharmaceuticals. In these mice, LoxP sites were integrated to excise the entire protein-coding region in exon 3 of the *Bmp2* gene. Generation of *Bmp4* conditional allele has been described [16,55]. The *Prx1::cre* transgene has been described [32].

### Generation of *Bmp2*, *Bmp4* double conditional mice and *Bmp2* conditional, *Bmp7* mutant mice.

*Bmp2* ^C/C^ animals were crossed with *Bmp4* ^C/C^ animals to generate *Bmp2* ^C/+^; *Bmp4* ^C/+^ animals. These animals were crossed with *Bmp2* ^C/C^ or *Bmp4* ^C/C^ animals to generate *Bmp2* ^C/C^; *Bmp4* ^C/+^ or *Bmp2* ^C/+^; *Bmp4* ^C/C^ animals, respectively. *Bmp2* ^C/C^; *Bmp4* ^C/+^ or *Bmp2* ^C/+^; *Bmp4* ^C/C^ animals were crossed with each other to generate *Bmp2* ^C/C^; *Bmp4* ^C/C^ animals. *Bmp2* ^C/C^; *Bmp4* ^C/C^ females were crossed with males bearing *Prx1::cre* transgene to generate *Bmp2^C/+^*; *Bmp4^C/+^*; *Prx1::cre* animals. *Bmp2^C/+^*; *Bmp4^C/+^*; *Prx1::cre* males were crossed with *Bmp2^C/C^*; *Bmp4^C/C^* females to generate *Bmp2^C/C^*; *Bmp4^C/C^*; *Prx1::cre* animals. *Bmp2^C/C^*; *Bmp4^C/C^* mice carrying floxed alleles of the *Bmp* genes without *Prx1::cre* are wild-type (WT). Genotyping of animals were done by PCR analyses with the primers (5′–3′) GTGTGGTCCACCGCATCAC (AHP2–9) and GGCAGACATTGTATCTCTAGG (AHP2–35) for *Bmp2* and AGACTCTTTAGTGAGCATTTTCAAC (No. 79 [order of primers tried]) and AGCCCAATTTCCACAACTTC (No. 80) for *Bmp4* following extraction of genomic DNA from embryonic or adult tail. For *Bmp2*, the floxed allele amplifies as a 545-bp product, while the wild-type allele amplifies as a 474-bp product. For *Bmp4,* the floxed allele amplifies as a 220-bp product, while the wild-type allele amplifies as a 180-bp product. Protocols utilized for mouse experiments were approved by the Harvard University Institutional Animal Care and Use Committee (Cliff Tabin #02735).

The strategy for generating a *Bmp2* conditional null in the *BMP7* ^−*/*−^ background is similar to the strategy used for generating the *Bmp2, Bmp4* double conditional allele with three important differences. First, the *Bmp7* ^−*/*−^ (strain kindly donated by Dr. Liz Robertson [12]) is not a conditional allele but a null mutation. Second*, Bmp7* ^−*/*−^ mice do not survive past birth due to kidney failure [12] and hence cannot be maintained as homozygous for the null allele. Third, *Bmp2* and *Bmp7* are on the same chromosome (Chromosome 2). To obtain *Bmp2^C/C^*, *Bmp7* ^−*/*−^; *Prx1::cre* we crossed *Bmp2^C/C^*, *Bmp7* ^−*/+*^ females with *Bmp2^C/+^*, *Bmp7* ^−*/+*^; *Prx1::cre* males. *Bmp2^C/C^*, *Bmp7* ^−*/*−^; *Prx1::cre* progeny were obtained at a rate slightly higher than 1 in 16. For genotyping of *Bmp7* locus we used the following set of primers: *Bmp7*_ExI_5′ (5′–3′) TCGCCTGCAGCAAGTGACCTCGGGTC; *Bmp7*_ExI_3′ (5′–3′) TAGGGGTAGGAGAAGCCCTGTCCGTCC and Beta-geo 5′ of 3′ (5′–3′) CTGCATACGCTTGATCCGGCTACCTGC. From a wild-type chromosome *Bmp7*_ExI_5′ and *Bmp7*_ExI_3′ amplifies an approximately 423-bp PCR product, while from the lacZ insertion mutant this pair fails to amplify any product. *Bmp7*_ExI_5′ and Beta-geo 5′ of 3′ (this primer is designed from the Beta-geo cassette) amplifies an approximately 1-kb PCR product from the lacZ inserted chromosome.

### Skeletal analysis.

Our protocol is an adaptation of the procedure described [56] previously. Newborn and older animals were killed using carbon dioxide. Skin, viscera, and adipose tissues were removed within 2 h after death, and samples were fixed in 95% ethanol for 5 d. Samples were then placed in acetone to remove residual fat for 2 d. For E13.5 embryos, skin was not removed. The dehydrated animals were stained with 0.015% w/v Alcian blue and 0.005% w/v Alizarin red in a 1:19 mixture of glacial acetic acid and 70% ethanol. The younger animals were stained for 5 h at 37 °C and then overnight at room temperature, while the newborns were stained overnight at 37 °C followed by 2 d at room temperature. The stained skeletons were kept in 1% KOH, with occasional change of solution, until cleared. The cleared skeletons were transferred into 100% glycerol and photographed.

### Acridine orange staining for apoptotic cells.

A working stock of 5 mg/ml acridine orange was diluted 1:10,000 in PBS. Dissected fresh embryos were transferred to the working solution of acridine orange and incubated for 30 min at 37 °C in the dark. Embryos were then washed twice in PBS for 5 min and viewed with a fluorescence microscope.

### Whole mount in situ hybridization.

In situ hybridization staining on whole embryos was performed as described [57]. For section in situ hybridizations, please see below.

### Histology, section in situ hybridizations, and immunohistochemistry.

For histology and section in situ hybridization, samples were fixed in 4% paraformaldehyde in PBS at 4 °C overnight. Newborn and older animals were then decalcified in Tris buffer containing 10% EDTA and 7.5% polyvinylpyrrolidone (pH 7.5) at 4 °C for 3 wks. Samples were then dehydrated through a graded ethanol series, cleared in xylene, and embedded in paraffin. 8μm sections were collected and stained with hematoxylin and eosin (H & E) or toluidine blue following standard procedure.

Section in situ hybridization with digoxigenin labeled probes was performed as described [57]. Section in situ hybridization with radiolabeled probes was performed as described [58] with a small modification. Briefly, sections were deparaffinized in xylene and rehydrated through a graded ethanol series. Sections were postfixed in 4% paraformaldehyde in PBS at room temperature for 15 min, digested with 10 μg/ml proteinase K at room temperature for 10 min, and again fixed in 4% paraformaldehyde at room temperature for 10 min and acetylated in the solution containing 0.2% hydrochloric acid, 0.1 M triethanol amine, and 0.25% acetic anhydride at room temperature for 10 min. Sections were then dehydrated in increasing concentrations of ethanol and air-dried. Hybridization was performed in a humidified chamber in a solution containing 50% formamide, 10% dextran sulfate, 1× Denhardt's solution, 0.6 M sodium chloride, 0.01 M Tris buffer (pH 7.5), 0.001 M EDTA, 0.05 M dithiothreitol, 0.25% SDS, 200 μg tRNA, and ^35^S-labeled cRNA probe at the final concentration of 5 × 10^6^ cpm/ml at 55 °C for 16 h. After hybridization, sections were washed with a solution containing 5× SSC and 10 mM dithiothreitol at 50 °C for 30 min, incubated in a solution containing 50% formamide, 2× SSC, and 10 mM dithiothreitol at 65 °C for 30 min, treated in a solution containing 10 μg/ml RNase A in TNE [0.01 M Tris buffer (pH 7.6), 0.5 M sodium chloride, and 0.001 M EDTA] at 37 °C for 30 min and then washed with 50% formamide, 2× SSC, and 10 mM dithiothreitol at 65 °C for an additional 30 min followed by two washes with 2× SSC/10 mM dithiothreitol at 65 °C for 30 min, and 0.1× SSC/10 mM dithiothreitol at 65 °C for 30 min.

To visualize the signal, sections were dipped into NTB-2 emulsion (Kodak, http://www.Kodak.com) and placed at 4 °C. After developing, sections were counterstained with H&E. The ^35^S-labeled cRNA probes were transcribed from plasmids encoding *type I collagen* [59], *runx2* [60], *osteopontin* [61], and *osterix* [46]. Sense and antisense probes were synthesized from linearized plasmids using a Riboprobe Combination System (Promega, http://www.promega.com).

## Supporting Information

Figure S1 Chondrogenic Differentiation in *Bmp2^C/C^*; *Bmp4^C/C^*; *Prx1::cre* Animals (A–C) In BMP2, BMP4–deficient limbs, there is only one zeugopod present, and it is often fused with the stylopod. All panels are from E17.5 *Bmp2^C/C^*; *Bmp4^C/C^*; *Prx1::cre* embryos. (A) Hematoxylin and eosin–stained section of the forelimb. Alcian blue– and Alizarin red–stained skeletons shown in (B) (forelimb) and (C) (hindlimb). Black arrow shows the fusion of the zeugopod and stylopod in BMP2, BMP4–deficient limbs.
(D and E) Acridine orange–stained limbs from E11.5 wild-type (D) and *Bmp2^C/C^*; *Bmp4^C/C^*; *Prx1::cre* embryos (E).
(F and G) Acridine orange–stained hindlimbs from E13.5 wild-type (F) and *Bmp2^C/C^*; *Bmp4^C/C^*; *Prx1::cre* (G) embryos.
(H–M) In the absence of BMP2 and BMP4, condensation begins in the limb normally. Whole mount in situ staining of *Sox9* mRNA in embryonic limb buds from E10.5 (H and I) and E11.5 (J–M) embryos. (H, J, and L) From wild-type embryos, (I, K, and M) from *Bmp2^C/C^*; *Bmp4^C/C^*; *Prx1::cre* embryos. (H, I, J, and K) Forelimbs, (L and M) hindlimbs.
(N and O) In situ hybridizations with *Col II* mRNA probes of sections derived from E12.5 wild-type (N) and *Bmp2^C/C^*; *Bmp4^C/C^*; *Prx1::cre* (O) embryos.
(P–S) *Bmp6* mRNA expression is not increased in E13.5 *Bmp2^C/C^*; *Bmp4^C/C^*; *Prx1::cre* cartilage. *Bmp6* in situ on sagittal sections from wild-type (Q) and *Bmp2^C/C^*; *Bmp4^C/C^*; *Prx1::cre* (S) embryonic forelimbs. (P and R) Bright field images of (Q) and (S), respectively.
(23 MB TIF)

Figure S2 Skeletal Differentiation Occurs Normally in Absence of BMP2 (A) Toluidine blue–stained section of newborn femurs from *Bmp2^C/C^* animals.
(B–D) Marker analyses. Sections of femurs from *Bmp2^C/C^* animals hybridized with *Col II* (B), *Col X* (C), and *Col I* (D) mRNA probes.
(E) Toluidine blue–stained section of newborn femurs from *Bmp2^C/C^*; *Prx1::cre* animals.
(F–H) Marker analyses. Sections of femurs from *Bmp2^C/C^*; *Prx1::cre* animals hybridized with *Col II* (F), *Col X* (G), and *Col I* (H) mRNA probes.
(15 MB TIF)

Figure S3 BMP2, BMP4–Deficient Limb Skeleton Is Resorbed Alcian blue– and Alizarin red–stained skeletons of forelimbs (A–D) and hindlimbs (E–F) from 1-wk-old (A, B, E, and F) and 3-wk-old (C and D) animals. (A, C, and E) Wild-type animals, (B, D, and F) *Bmp2^C/C^*; *Bmp4^C/C^*; *Prx1::cre* animals. The limbs of *Bmp2^C/C^*; *Bmp4^C/C^*; *Prx1::cre* (H) animals are severely defective compared to a wild-type (G) animal at 1 wk of age.
Please note that the proximal part of the femur is present at 1 wk of age but missing at 3 wks (refer to Figure 6L).
(12 MB TIF)

Figure S4 Osteogenesis Occurs in Mice Lacking Three of Four Alleles of Bmp2 and Bmp4 Combined Toluidine blue–stained sections of femurs from adult control, *Bmp2^C/+^; Bmp4^C/C^*; *Prx1::cre,* and *Bmp2^C/C^*; *Bmp4^C/+^*; *Prx1::cre* animals.
(11 MB TIF)

## Abbreviations

AER: apical ectodermal ridge
BMP: bone morphogenetic protein
E: embryonic day
FGF: fibroblast growth factor
SHH: Sonic Hedgehog
*Shh*: *sonic hedgehog*
ZPA: zone of polarizing activity
